# Norepinephrine inhibits CD8^+^ T-cell infiltration and function, inducing anti-PD-1 mAb resistance in lung adenocarcinoma

**DOI:** 10.1038/s41416-022-02132-7

**Published:** 2023-01-16

**Authors:** Qishun Geng, Lifeng Li, Zhibo Shen, Yuanyuan Zheng, Longhao Wang, Ruyue Xue, Wenhua Xue, Mengle Peng, Jie Zhao

**Affiliations:** 1 Internet Medical and System Applications of National Engineering Laboratory, The First Affiliated Hospital of Zhengzhou University, 450052 Zhengzhou, Henan China; 2 Department of Pharmacy, The First Affiliated Hospital of Zhengzhou University, 450052 Zhengzhou, China; 3 China-Japan Friendship Hospital (Institute of Clinical Medical Sciences), Chinese Academy of Medical Sciences & Peking Union Medical College, 100193 Beijing, China

**Keywords:** Cancer immunotherapy, Drug development

## Abstract

**Background:**

Mental stress-induced neurotransmitters can affect the immune system in various ways. Therefore, a better understanding of the role of neurotransmitters in the tumour immune microenvironment is expected to promote the development of novel anti-tumour therapies.

**Methods:**

In this study, we analysed the plasma levels of neurotransmitters in anti-programmed cell death protein 1 (PD-1) monoclonal antibody (mAb)-resistance patients and sensitive patients, to identify significantly different neurotransmitters. Subsequently, animal experiments and experiments in vitro were used to reveal the specific mechanism of norepinephrine’s (NE) effect on immunotherapy.

**Results:**

The plasma NE levels were higher in anti-PD-1 mAb-resistance patients, which may be the main cause of anti-PD-1 mAb resistance. Then, from the perspective of the immunosuppressive microenvironment to explore the specific mechanism of NE-induced anti-PD-1 mAb resistance, we found that NE can affect the secretion of C-X-C Motif Chemokine Ligand 9 (CXCL9) and adenosine (ADO) in tumour cells, thereby inhibiting chemotaxis and function of CD8^+^ T cells. Notably, the WNT7A/β-catenin signalling pathway plays a crucial role in this progression.

**Conclusion:**

NE can affect the secretion of CXCL9 and ADO in tumour cells, thereby inhibiting chemotaxis and the function of CD8^+^ T cells and inducing anti-PD-1 mAb resistance in lung adenocarcinoma (LUAD).

## Introduction

An increasing number of studies have shown that stress response pathways, including the hypothalamic-pituitary-adrenal (HPA) axis and the sympathetic nervous system (SNS), are closely related to the occurrence and development of tumours caused by mental stress [1–3]. Clinical studies have found that stress factors play a crucial role in the occurrence and development of tumours, which can increase tumour risk and affect tumour treatment [4]. Neurotransmitters produced by mental stress can directly or indirectly modulate tumour-related pathways [5]; these are neurosecretory substances, including dopamine, norepinephrine (NE), adrenaline, 5-hydroxytryptophan (5-HT), gamma-aminobutyric acid (GABA), etc. Neurotransmitters bind to their receptors to stimulate or inhibit neuronal function, thereby regulating physiological changes of effector cells. Over the past few decades, numerous studies have revealed the regulatory roles of neurotransmitters in the physiological and pathological functions of tissues and organs [6]. Increased studies reported nerve signalling can contribute to tumour progression; thus, a recent study used genetic engineering to induce the expression of long-acting sodium channels in intratumoral adrenergic nerves. Expression of these long-acting channels increased adrenergic neuronal activity and intratumoral levels of NE, resulting in the accelerated growth of orthotopic and carcinogen-induced mammary tumours in mice [7]. NE and corticosterone levels were abnormally increased in the peripheral blood, abdominal organs and tumour tissues of animals under chronic stress, which could stimulate the rapid growth of transplanted tumours [8]. All of these studies suggest that neurotransmitters induce uncontrolled proliferation and metastasis of tumours by activating tumour-promoting signalling pathways.

Research on the mutual regulation between the nervous system and the immune system has developed rapidly in recent years. The nervous system regulates the function of the immune system through extensive peripheral neurites and neurotransmitters; the immune system acts on the neuroendocrine system through a variety of cytokines and hormone-like substances [9]. This bidirectional complex interaction enables mutual regulation within or between the two systems to jointly affect tumour progression. Researchers have conducted extensive studies in both animal models and human tumour patients, which have shown that mental stress can affect the activity and distribution of adrenergic receptors, glucocorticoid receptors and prostaglandin receptors on most immune cells [10]. Neurotransmitters induced by mental stress can affect the immune system in a variety of ways, some of which lead to tumour immune escape [11, 12]. Cao et al. found that chronic restraint stress promotes the mobilisation and recruitment of myeloid-derived suppressor cells through β-adrenergic-activated CXCL5-CXCR2-Erk signalling cascades [13]. Several studies have also shown that stress can enhance the immune evasion of tumour cells by increasing immunosuppressive cells such as regulatory T cells (Tregs) and regulatory B cells (Bregs), which are associated with poor patient outcomes and increased mortality [14, 15]. These studies have shown that tumour cells use neurotransmitter-initiated signalling pathways to influence immune cells in the tumour microenvironment, thereby promoting tumour progression. Therefore, a better understanding of the potential functions of neurotransmitters in tumorigenesis, immunity and inflammation is required to develop novel anti-tumour therapies, while facilitating immune synergistic therapy [5].

In clinical practice, we found that most anti-PD-1 mAb-resistance patients showed more serious mental stress, which affected the secretion of neurotransmitters. Based on this clinical phenomenon, this study aims to find the relationship between neurotransmitters and anti-PD-1 mAb resistance. Through animal and cell experiments, we found that NE can affect the secretion of the chemokine CXCL9 and the immunosuppressive metabolite ADO in tumour cells by regulating the WNT7A/β-catenin signalling pathway, thereby inhibiting chemotaxis and function of CD8^+^ T cells. Our study elucidates the reasons for anti-PD-1 mAb resistance in LUAD patients from the perspective of neurotransmitters, which indicates that central nervous system (CNS) drugs can play an important role in alleviating anti-PD-1 mAb resistance. More importantly, this study further emphasises the significance of CNS drugs combined with anti-PD-1 mAb in improving the survival rate and life quality of tumour patients. The finding not only guides clinical therapeutic strategies but also facilitates synergistic immunotherapy.

## Methods

### Cell culture and cell transfection

The human LUAD cell lines A549 and H3122 were acquired from the Chinese Academy of Sciences (Shanghai, China). The cells were separately maintained in DMEM/RPMI1640 with 10% FBS (Thermo Fisher Scientific, USA) and 1% penicillin–streptomycin (Thermo Fisher Scientific, USA), which were cultured at 37 °C, 5% CO_2_ and saturated humidity. A549 and H3122 were used in this study because they both express β_2_ adrenoceptors and are the most common cell lines in lung adenocarcinoma [16]. To investigate the effect of NE on tumour cells, cells were treated with 10 uM NE for 24 h and the related protein expression changes were detected.

A549 and H3122 cells with stable CD39/WNT7A/β-catenin knockdown was generated using lentivirus-mediated knockdown of CD39/WNT7A/β-catenin. Lentiviral vectors encoding shRNA targeting CD39/WNT7A/β-catenin or scramble as the negative control (NC) were purchased from Tsingke Biotechnology Co. Ltd. (Shanghai, China). The efficiency of gene silencing was assayed by western blotting. The target sequences were shown as follows: shWNT7A: GCGTTCACCTACGCCATCATT, shCD39: AAGTACCTGAGTGAATACTGC, shβ-catenin: GGATGTGGATACCTCCCAAG, shCXCL9: CATCATCTTCCTGGAGCAGTG.

### Cell isolation and sorting

Peripheral blood mononuclear cells (PBMCs) were isolated from the blood samples by Ficoll–Hypaque density gradient centrifugation. CD8^+^ T cells were enriched using an anti-CD8 MACS magnetic sorting system (Miltenyi Biotec, Germany) to a purity of more than 95%. The purified CD8^+^ T lymphocytes were used for the transwell assay.

### Real-time quantitative PCR (RT-qPCR)

The TRIzol reagent (Invitrogen, Carlsbad, CA, USA) was used to extract RNA from cells. Complementary DNA (cDNA) was synthesised from total RNA using PrimeScript RT Reagent Kit (Takara, Dalian, China), and PCR was performed using the SYBR Green RT-PCR Kit (AG11701, ACCURATE BIOTECHNOLOGY, HUNAN, Co., Ltd.). GAPDH served as an internal control. PCR was performed on the StepOne Plus Real-Time PCR System (Applied Biosystems, Foster City, CA, USA), and the data were analysed using the 2-ΔΔCT method. Details of the primer sequences are listed in Table 1. In addition, chemokines expression was determined using SYBR Green quantitative PCR and the RT Chemokines & Receptors PCR Array plate (WC-MRNA0033-H, GENE Biotech, Shanghai, China) according to the kit introduction.

**Table 1:** The sequences of primers used for RT-qPCR.

Gene	Forward sequence (5′-3′)	Reverse sequence (5′-3′)
Human		
WNT7A	ACGGCCTGTGCTTCTTCTTA	CCGGCTAACATGGATGAGAT
IFN-r	GAGTGTGACCATCAAGG	CATGTATTGCTTTGCGTTGG
CXCL9	CCACCGAGATCCTTATCGAA	CTAACCGACTTGGCTGCTTC
ß-actin	CCTTCCTGGGCATGGAGTC	TGATCTTCATTGTGCTGGGTG
Mouse		
Wnt1	GGTTTCTACTACGTTGCTACTGG	GGAATCCGTCAACAGGTTCGT
Wnt2	CTCGGTGGAATCTGGCTCTG	CACATTGTCACACATCACCCT
Wnt3A	CTCCTCTCGGATACCTCTTAGTG	GCATGATCTCCACGTAGTTCCTG
Wnt4	AGACGTGCGAGAAACTCAAAG	GGAACTGGTATTGGCACTCCT
Wnt5A	GGTGAGGGACTGGAAGTTGC	GGAGCAGATGTTTATTGCCTT
Wnt6	GCAAGACTGGGGGTTCGAG	CCTGACAACCACACTGTAGGAG
Wnt7A	CCTTGTTGCGCTTGTTCTCC	GGCGGGGCAATCCACATAG
Gapdh	AAATGGTGAAGGTCGGTGTGA AC	CAACAATCTCCACTTTGCCACTG
Wnt10A	GCTCAACGCCAACACAGTG	CGAAAACCTCGGCTGAAGATG

### Western blotting

Cells were lysed using radioimmunoprecipitation assay (RIPA) buffer (Beyotime, Shanghai, China) supplemented with a protease inhibitor cocktail (Thermo Scientific, Waltham, MA, USA) and phenylmethanesulfonyl fluoride (PMSF, Beyotime, Shanghai, China). The bicinchoninic acid (BCA) method was used to measure protein concentration. Cell lysates (40 μg) were subjected to sodium dodecyl sulfate-polyacrylamide gelelectrophoresis (SDS-PAGE), transferred to polyvinyldene fluoride (PVDF) membrane (Sigma, St Louis, MO, USA). The membrane was incubated with specific antibodies against β-catenin (1:1000 dilution; Affinity, DF3845), WNT7A (1:1000 dilution; abcam, ab274321), CD39 (1:1000 dilution; ABclonal, A3778), CD73 (1:1000 dilution; Proteintech, 12231-1-AP) and CXCL9 (1:1000 dilution; ABclonal, A1864), followed by incubation with horseradish peroxidase (HRP)-conjugated anti-IgG (Beyotime, Shanghai, China). Autoradiograms were densitometrically quantified using Quantity One software (Bio-Rad), with β-actin/GAPDH (Beyotime, Shanghai, China) serving as internal controls.

### Enzyme-linked immunosorbent assay (ELISA)

NE and other neurotransmitters in serum were quantified by ELISA Kit (Cusabio, Wuhan, China) according to the manufacturer’s protocol. The levels of CXCL9 and ADO were also detected using the Human CXCL9 PicoKine ELISA Kit (Boster, Pleasanton, CA, USA) and ADO assay Kit (Abcam, ab211094, USA), respectively, according to the manufacturer’s instructions.

### Flow cytometric analysis

Cells were stained with FITC anti-human CD8 Antibody, PE/Cyanine7 anti-mouse CD8 Antibody, PE/Cyanine7 anti-human IFN-γ Antibody and PerCP/Cyanine5.5 anti-mouse IFN-γ Antibody according to the manufacturer’s instructions (BioLegend, USA). Among them, IFN-γwere used for intracellular staining. Then, the samples were collected by flow cytometry (BD FACSCanto II, USA) according to the manufacturer’s instructions, and data analysis was conducted with FlowJO software (version 10.0, Tree Star, Inc. Ashland, OR, USA).

### Immunohistochemistry

For histological examination, tumour tissue samples were embedded in paraffin blocks and cut into 3 μm thick sections, which were made into tumour arrays. According to the previous description, tumour arrays were stained and scored [17].

### Transcriptome analysis

Total RNA from PBMCs and whole blood was isolated, respectively, and used for RNA-seq analysis. Sample processing and data analysis were performed as previously described [18]. RNA-Seq data are available as GEO accession number GSE218402.

### Metabolomic analysis

The blood of the mouse was used for the metabolomic analysis. Sample processing and data analysis were carried out according to previous studies [18].

### Animal experiments

To generate a subcutaneous tumour mouse model, all male C57BL/6 mice (4–6 weeks old and weighing 18–20 g) were purchased from Beijing Vital River Laboratory Animal Technology Co., Ltd (China). All animals were fed under constantly standard environmental conditions (23 ± 1 °C, 55 ± 5% humidity and a 12/12 h light/dark cycle) for 1 week before the experiments.

The ethical approval for the animal experiments was obtained from the Ethics Committee of the First Affiliated Hospital of Zhengzhou University. All animal studies were performed according to the guidelines provided by the Animal Ethics Committee of the Institute of Zoology, Chinese Academy of Sciences. After adjusting to the environment, 35 mice were subcutaneously inoculated with 1 × 10^6^ LLC cells. When the tumour volume reached≈100 mm^3^, the mice were divided into seven groups: control (CTRL), NE, NE + propranolol (Prop), anti-PD-1 mAb, anti-PD-1 mAb+Prop, anti-PD-1 mAb +NE and anti-PD-1 mAb+NE + Prop. Based on previous experiments and related literature [19, 20], norepinephrine was injected into mice at a dose of 2 mg/kg; mice received 10 mg/kg propranolol (P0884, Sigma–Aldrich) by i.p. injection; anti-mouse PD-1 antibody (RMP1-14) was purchased from BioXCell, which also was injected into mice at a dose of 10 mg/kg. According to the dosage required by each group of mice, it was dissolved in 100 ul PBS and administered by intraperitoneal injection. The control mice received 100 μl PBS. From the day of grouping, the mice were intervened in groups, once every 2 days for a total of seven times. After 15 days, because the tumour size reached 2000 mm^3^ in size, the mice were sacrificed in the method of spinal cord dislocation according to the guidelines. Then, the tumour is weighed and samples such as plasma are collected.

### Human samples

Blood samples were obtained from 20 patients with LUAD at the First Affiliated Hospital of Zhengzhou University. These patients were subjected to routine laboratory diagnosis, who have used anti-PD-1 mAb. Written informed consent was obtained from all the patients. The Ethical approval for the experiments was obtained from the Ethics Committee of the First Affiliated Hospital of Zhengzhou University (2020-KY-0379-002).

### Statistical analysis

Statistical analyses were performed using the GraphPad Prism software (GraphPad Software, San Diego, CA, USA). R version 4.0.4 software (Institute for Statistics and Mathematics, Vienna, Austria; https://www.r-project.org) and SPSS 24.0 (IBM, NY, USA) were also used for statistical analyses. The student’s *t*-test was used for comparisons between the groups. All experiments were repeated three times. Measurement data were expressed as mean ± standard deviation, and *P* < 0.05 indicated statistical significance.

## Results

### NE is capable of causing the anti-PD-1 mAb resistance during the treatment of LUAD

**Fig. 1:**
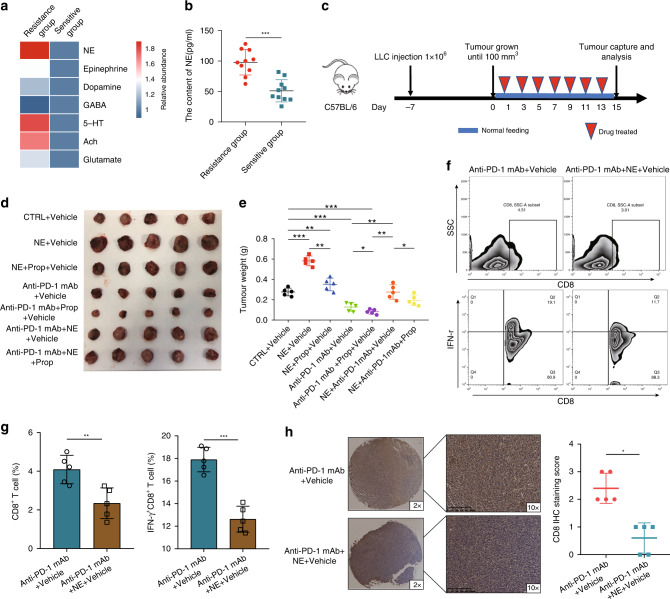
Norepinephrine (NE) is capable of causing the anti-PD-1 mAb resistance in the treatment of lung adenocarcinoma (LUAD). **a** The plasma levels of neurotransmitters in LUAD patients; **b** the plasma levels of NE were higher in the resistance group; **c** a scheme of the animal experiment; the tumour size (**d**) and weight (**e**) of mice in the different groups; **f**, **g** the proportion and function of CD8^+^ T cells and IFN-γ^+^ CD8^+^ T cells in tumour tissue of the anti-PD-1 mAb+vehicle and anti-PD-1 mAb+NE + vehicle groups; **h** the CD8 expression level in tumours was detected using immunohistochemistry. *P < 0.05, **P < 0.01, ***P < 0.001.

During the period of 2019–2021, we collected plasma from 20 LUAD patients treated with anti-PD-1 mAb. According to the iRECIST evaluation criteria, the LUAD patients were divided into two groups: the resistance group (*n* = 10) and the sensitive group (*n* = 10) [21]. Then, the levels of neurotransmitters (5-HT, NE, GABA, epinephrine, acetylcholine, glutamate and dopamine) in the plasma of LUAD patients were detected by ELISA kit, the fold change in neurotransmitter content compared to the resistance group is shown in Fig. 1a. And we found that the plasma levels of NE in the resistance group were higher (Fig. 1b). Therefore, we speculated that NE may cause anti-PD-1 mAb resistance in patients with LUAD. To verify this hypothesis, we constructed a subcutaneous tumour mouse model (Fig. 1c). By comparing the tumour weight and size of the CTRL, NE, anti-PD-1 mAb and anti-PD-1 mAb + NE groups, we found that NE significantly reduced the therapeutic effect of anti-PD-1 mAb (Fig. 1d, e).

The main reasons for anti-PD-1 mAb resistance include insufficient tumour immunogenicity, irreversible T-cell depletion, immunosuppressive microenvironment and oncogene mutations [22]. Among these, the immunosuppressive microenvironment has received special attention. The main reasons are as follows: the immunosuppressive microenvironment is the most common cause of anti-PD-1 mAb resistance; previous studies have shown that neurotransmitters can mediate tumour progression by regulating the tumour immune microenvironment [23]. To understand the effect of NE on the tumour immune microenvironment, the proportion and function of immune cells in tumours were detected by flow cytometry. We found that the proportion and function of CD8^+^ T cells were significantly reduced in the anti-PD-1 mAb + NE group compared to those in the anti-PD-1 mAb group (Fig. 1f, g). In addition, immunohistochemical analyses indicated that CD8 expression in the anti-PD-1 mAb + NE group was significantly reduced (Fig. 1h). These results show that NE is capable of causing the anti-PD-1 mAb resistance during LUAD treatment by reducing the proportion and function of CD8^+^ T cells.

### NE inhibits the secretion of CXCL9 by tumour cells, leading to reduced chemotaxis of CD8^+^ T cells in the tumour microenvironment

**Fig. 2:**
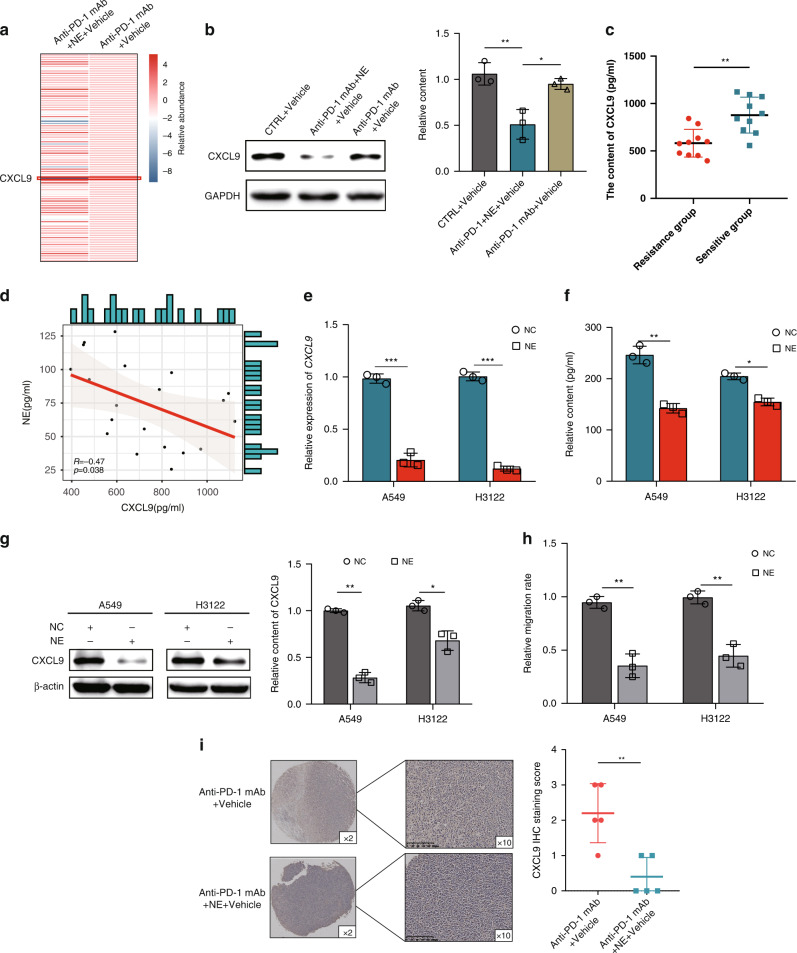
Norepinephrine (NE) inhibits the secretion of CXCL9 by tumour cells, leading to reduced chemotaxis of CD8^+^ T cells in tumour microenvironment. **a** The expression levels of common chemokines and receptors were detected by PCR array; **b** the expression of CXCL9 in the tumours of CTRL + Vehicle, NE + anti-PD-1 mAb + Vehicle and anti-PD-1 mAb + Vehicle groups was detected by western blotting; **c** the plasma content of CXCL9 was also lower in the resistance group; **d** the plasma content of CXCL9 was greatly correlated with the content of NE; the CXCL9 mRNA/protein expression levels in A549 and H3122 cells were detected by RT-qPCR (**e**), ELISA (**f**) and western blotting (**g**); **h** the quantification of CD8^+^ T-cell migration activity was measured and compared; **i** the CXCL9 expression level in tumours was detected in the anti-PD-1 mAb+vehicle and anti-PD-1 mAb+NE + vehicle groups using immunohistochemistry. *P < 0.05, **P < 0.01.

By comparing the proportion and function of immune cells in animal model tumours, we found that NE can significantly reduce the proportion of CD8^+^ T cells, suggesting that NE can affect the chemotaxis of CD8^+^ T cells in the tumour microenvironment. Firstly, RNA was extracted from the tumour tissues of two groups of mice, anti-PD-1 mAb + Vehicle and NE + anti-PD-1 mAb + Vehicle, and the expression levels of common chemokines and receptors were detected by PCR array. By comparing the differentially expressed genes (DEGs) between the two groups, it was found that there were significant differences in CXCL9 (Fig. 2a, Supplementary Table 1). CXCL9, also known as monokine induced by IFN-γ, is a small molecular weight cytokine belonging to the CXC chemokine family, which is associated with CD8^+^ T-cell infiltration in solid tumours [24]. In addition, the expression of CXCL9 in tumour tissues of CTRL + Vehicle, NE + anti-PD-1 mAb + Vehicle and anti-PD-1 mAb + Vehicle groups was further detected by western blot. The results showed that the expression of CXCL9 in tumour tissues of the NE + anti-PD-1 mAb + Vehicle group was significantly lower than that in the CTRL + Vehicle and anti-PD-1 mAb + Vehicle groups (Fig. 2b). These results suggest that NE can inhibit the expression of CXCL9 in tumour tissues. Notably, the plasma content of CXCL9 was also lower in the resistance group (Fig. 2c). Moreover, the content of CXCL9 in the patient’s plasma was significantly correlated with NE content (Fig. 2d).

To further verify the effect of NE on the expression of CXCL9 in tumour tissues, we stimulated LUAD cell lines (A549 and H3122) with NE and detected the changes in CXCL9 mRNA levels by RT-qPCR. The results showed that NE significantly reduce the CXCL9 mRNA expression level in A549 and H3122 cell lines (Fig. 2e). Then, the protein level of CXCL9 was detected by ELISA (Fig. 2f) and western blot (Fig. 2g). The results also showed that the expression of CXCL9 in A549 and H3122 cell lines decreased after NE stimulation. These results all suggest that NE can inhibit the expression of CXCL9 in LUAD cells. In order to investigate whether NE-treated A549/H3122 medium affects CD8^+^ T-cell migration in vitro, supernatants of NE-treated tumour cells (after 24 h) were loaded into the down wells of a 0.3 μm transwell plate to attract CD8^+^ T-cell migration, which showed that NE-treated tumour cell supernatants significantly reduced CD8^+^ T-cell migration (Fig. 2h). To exclude a direct effect of NE on CD8^+^ T cells, CD8^+^ T cells treated with a medium containing the same amount of NE was performed. The result indicates NE does not directly affect CD8^+^ T-cell migration (Supplementary Fig. 1A). In addition, we transfected tumour cells with CXCL9-shRNA lentivirus or scramble-shRNA lentivirus (NC) to further determine the role of CXCL9. Western blotting confirmed the efficiency of gene silencing (Supplementary Fig. 1B). Compared with the supernatants from NC-treated A549^shCXCL9^/H3122^shCXCL9^, the CM from NE-treated A549^shCXCL9^/H3122^shCXCL9^ cells did not influence the CD8^+^ T-cell migration (Supplementary Fig. 1C). The results of the immunohistochemical analysis also showed that the expression of CXCL9 in the anti-PD-1 mAb + NE group was significantly reduced, which was consistent with the expression of CD8 in the anti-PD-1 mAb + NE group (Fig. 2i). These results further confirm that NE inhibits the secretion of CXCL9 by tumour cells, resulting in decreased CD8^+^ T-cell chemotaxis in the tumour microenvironment.

### NE promotes ADO production by tumour cells

**Fig. 3:**
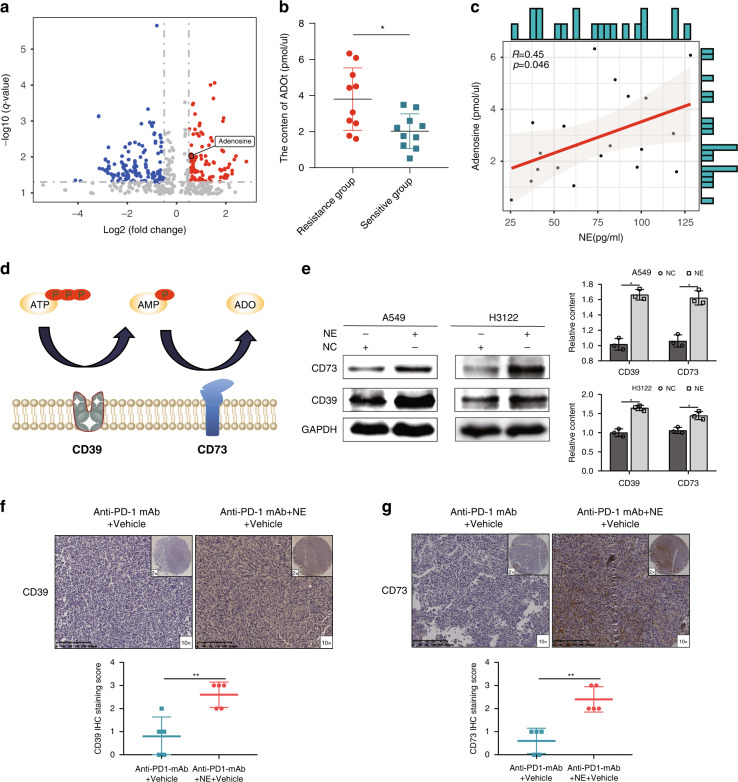
Norepinephrine (NE) promotes adenosine (ADO) production by tumour cell. **a** The volcano plots of the differential metabolites for the anti-PD-1 mAb+NE + vehicle group vs. the anti-PD-1 mAb + vehicle group; **b** the plasma content of ADO in the resistant and sensitive groups; **c** the plasma content of ADO was correlated with the content of NE; **d** the process of ADO metabolism; **e** the protein levels of CD39 and CD73 in lung adenocarcinoma (LUAD) cell lines (A549 and H3122) after NE stimulation; **f**, **g** the CD39 and CD73 expression level in tumours were detected in the anti-PD-1 mAb+vehicle and anti-PD-1 mAb+NE + vehicle groups using immunohistochemistry. *P < 0.05, **P < 0.01.

Previous studies have shown that cancer metabolism plays a crucial role not only in tumour signalling sustaining tumorigenesis and progression but also in the expression of immune molecules through the release of metabolites, which has broader implications in regulating tumour immune responses [25]. To investigate the metabolic differences caused by NE, untargeted metabolomics was used to detect the content of metabolites in the plasma of anti-PD-1 mAb + Vehicle and NE + anti-PD-1 mAb + Vehicle mice. The data of each sample processed by Compound Discovery software were subjected to principal component analysis (PCA) using SIMCA software. QC samples were held tightly together in both positive and negative ion modes, demonstrating the robustness, repeatability and reliability of the analytical method (Supplementary Fig. 2A, B). In addition, the PCA results showed there are great differences between the two groups, indicating NE can cause significant changes in plasma metabolite. To obtain the differential metabolites, we established an orthogonal partial least-squares discrimination analysis (OPLS-DA) model (Supplementary Fig. 2C, F). The separation between the two groups in the model’s scatter plot was very clear, showing that the mice in the two groups had significant differences in endogenous metabolites. After 200 permutation tests, the R2Y and Q2 values of the model were obtained (positive, R2Y = 1, Q2 = 0.734; negative, R2Y = 1, Q2 = 0.74), indicating that the model is stable and reliable, without over-fitting and has high prediction ability (Supplementary Fig. 2D, G). Based on the OPLS-DA model, the VIP values of the metabolites were obtained (Supplementary Fig. 2E, H), and the *P*-values and log2(fold change) values of the metabolites were obtained by *t*-test. Then, with VIP > 1.2, *P* < 0.05 and |log2(FC) | > 0.5 as screening conditions, the differential metabolites were obtained (Fig. 3a, Supplementary Table 2). Among them, adenosine (ADO) has received the most attention. Compared with the mice in anti-PD-1 mAb + Vehicle, the content of ADO was increased in the NE + anti-PD-1 mAb + Vehicle group. Although among these metabolites, ADO variability was not the most pronounced. However, by querying the relationship between metabolites and immunity, we found that ADO was significantly associated with immunity. ADO is an immunosuppressive metabolite produced at high levels in the tumour microenvironment, which can potently suppress immune responses through A2a receptors expressed on immune cells [26].

Subsequently, ELISA was used to detect the ADO content in the plasma of the resistance and sensitive groups, which showed that the plasma ADO content of the resistance group was greatly increased (Fig. 3b). Moreover, the ADO content in the patient’s plasma was significantly correlated with the content of NE (Fig. 3c). Previous studies have shown that CD39 and CD73 expression is an important factor in ADO production [27] (Fig. 3d). To further prove that NE can affect ADO production, we stimulated LUAD cell lines (A549 and H3122) with NE, and detected the protein levels of CD39 and CD73 using western blot. The results showed that the expression levels of CD39 and CD73 markedly increased after NE stimulation (Fig. 3e). In addition, immunohistochemical analysis showed that the expression of CD39 and CD73 in the anti-PD-1 mAb + NE group was also higher (Fig. 3f, g). These results further confirmed that NE promoted the ADO production by increasing the expression of CD39 and CD73 in tumour cells.

### The ADO produced by tumour cells can affect CD8^+^ T-cell function

**Fig. 4:**
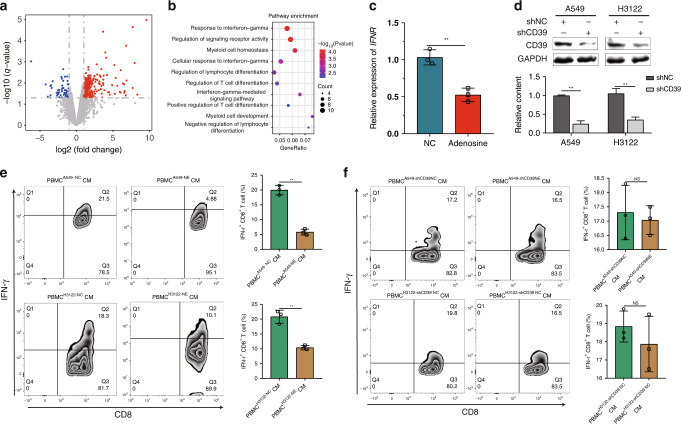
The adenosine (ADO) produced by tumour cells can affect CD8^+^ T-cell function. **a** The volcano plots of the differentially expressed genes (DEGs) between the ADO and the control groups; **b** the KEGG pathway enrichment analysis of the DEGs; **c** the mRNA expression of IFN-γ in PBMCs stimulated by ADO; **d** western blotting confirmed the efficiency of CD39 silencing; **e** compared with the conditioned medium (CM) from negative control (NC) -treated A549/H3122 cells, the CM from Norepinephrine (NE)-treated A549/H3122 cells was able to significantly reduce IFN-γ^+^ CD8^+^ T-cell rate in PBMCs; **f** compared with the CM from NC-treated A549^shCD39^/H3122^shCD39^ cells, the CM from NE-treated A549^shCD39^/H3122^shCD39^ cells did not reduce the IFN-γ^+^ CD8^+^ T-cell rate in PBMCs. *P < 0.05, **P < 0.01.

Several studies have shown that inhibiting ADO production or antagonising ADO receptors can promote antitumor immunity through a variety of mechanisms, including enhancing the function of T cells, inhibiting the pro-tumorigenic effects of myeloid cells and other immune regulatory cells, and promoting antigen presentation [28]. To explore whether ADO affects immune cell function, we treated PBMCs with 30 uM ADO for 24 h, which were subjected to transcriptome sequencing. Based on the filter of |log2(FC) | > 1.0 and *P* < 0.05, a total of 323 DEGs were obtained by differential analysis between the ADO group and the control group (Fig. 4a). In order to understand the impact of ADO on biological functions, we performed Kyoto Encyclopedia of Genes and Genomes (KEGG) pathway enrichment analysis of these DEGs (Fig. 4b). The DEGs were highly enriched in the following functions and pathways: response to IFN-γ, myeloid cell homoeostasis, cellular response to IFN-γ, regulation of lymphocyte differentiation, regulation of T-cell differentiation, IFN-γ-mediated signalling pathway, etc. These pathways are mainly related to immunity, further confirming that ADO participates in the regulation of immune functions. In particular, multiple signalling pathways are associated with IFN-γ. Therefore, we detected the effect of ADO on the expression of IFN-γ in PBMCs by RT-qPCR, which showed that ADO significantly reduced the mRNA expression of IFN-γ (Fig. 4c).

Considering the following reasons: (1) Animal experiments showed that NE can inhibit the function of CD8^+^ T cells; (2) NE can promote the production of ADO; (3) ADO can inhibit the expression of IFN-γ in PBMCs, we speculated that NE could promote ADO production in tumour cells, thereby inhibiting the IFN-γ function of CD8^+^ T cells. To test this hypothesis, conditioned medium (CM) from NC/NE-treated A549/H3122 cells were collected to culture with PBMCs. Compared with the CM from NC-treated A549/H3122 cells, the CM from NE-treated A549/H3122 cells significantly reduced the IFN-γ + CD8 + T-cell rate in PBMCs in vitro (Fig. 4e). To exclude a direct effect of NE on the IFN-γ function of CD8^+^ T cells, PBMCs treated with medium containing the same amount of NE was performed, indicating medium (DMEM/RPMI1640)-NE does not directly affect the IFN-γ function of CD8^+^ T cells (Supplementary Fig. 3A). To further determine the role of ADO in NE-stimulated tumour cells in suppressing the IFN-γ function of CD8^+^ T cells, we transfected tumour cells with CD39-shRNA lentivirus or scramble-shRNA lentivirus (NC). Western blotting confirmed the efficiency of gene silencing (Fig. 4d). The CMs from NC/NE-treated A549^shCD39^ /H3122^shCD39^ cells were collected and cultured with PBMCs. Compared with the CM from NC-treated A549^shCD39^/H3122^shCD39^, the CM from NE-treated A549^shCD39^/H3122^shCD39^ cells did not reduce the IFN-γ^+^ CD8^+^ T-cell rate in PBMCs (Fig. 4f). While, compared with the CM from NC-treated A549^shNC^/H3122^shNC^, the CM from NE-treated A549^shNC^/H3122^shNC^ cells reduced the IFN-γ^+^ CD8^+^ T-cell rate in PBMCs (Supplementary Fig. 3B). These results further confirmed that NE inhibited the IFN-γ function of CD8^+^ T cells by promoting ADO production in tumour cells.

### NE regulates the secretion of CXCL9 and ADO by WNT7A/β-catenin signalling in LUAD cells

**Fig. 5:**
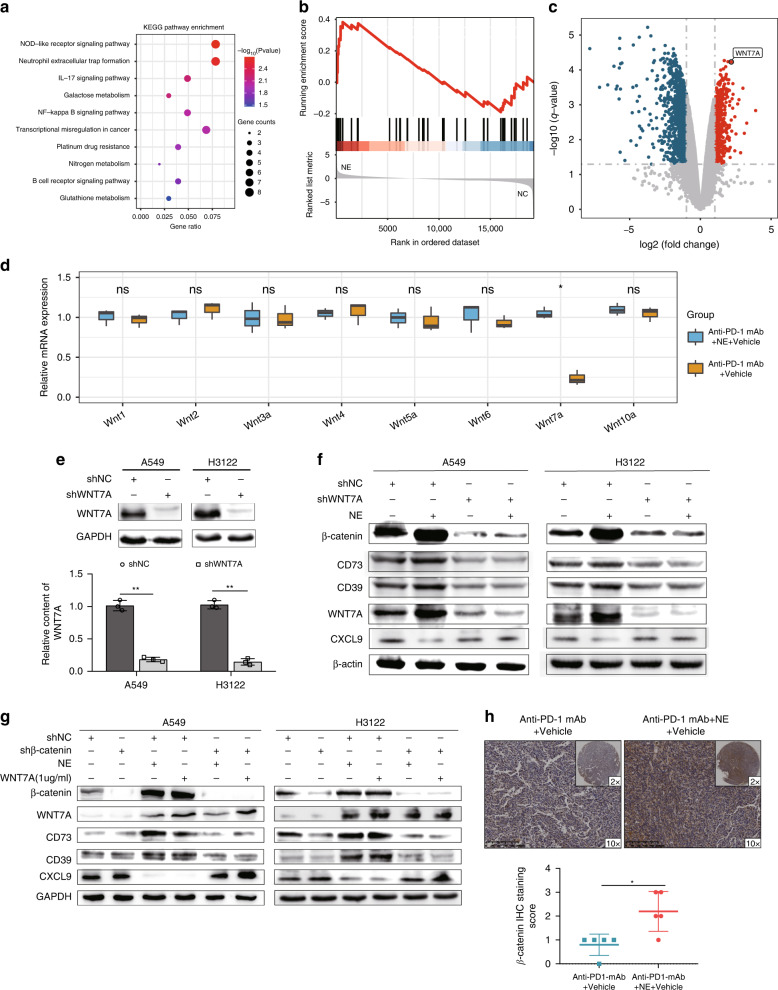
Norepinephrine (NE) regulates the secretion of CXCL9 and adenosine (ADO) by WNT7A/β-catenin signalling in lung adenocarcinoma (LUAD) cells. **a** The KEGG pathway enrichment analysis of the differentially expressed genes (DEGs); **b** the GSEA between the NE and the control (NC) groups; **c** the volcano plots of the DEGs; **d** the expression levels of Wnt1, Wnt2, Wnt3a, Wnt4, Wnt5a, Wnt6, Wnt7a and Wnt10a in the tumour tissues of the anti-PD-1 mAb + Vehicle and NE + anti-PD-1 mAb + Vehicle groups; **e** western blotting confirmed the efficiency of WNT7A silencing; **f** downregulation of WNT7A by shRNA significantly antagonised NE-induced β-catenin, CD39 and CD73 expression, as well as NE-inhibited CXCL9 expression in LUAD cells; **g** downregulation of β-catenin by shRNA greatly antagonised NE/WNT7A-induced CD39 and CD73 expression, as well as NE/WNT7A-inhibited CXCL9 expression in LUAD cells; **h** the β-catenin expression level in tumours was detected in the anti-PD-1 mAb + vehicle group and anti-PD-1 mAb+NE + vehicle group using immunohistochemistry. *P < 0.05, **P < 0.01.

In order to further study the mechanism of NE-induced changes of CXCL9 and ADO secretion in tumour cells, we used transcriptome sequencing to detect gene expression levels in A549 cells after NE stimulation. After integrating the transcriptome sequencing data, KEGG pathway enrichment analysis was performed. Significantly enriched signalling pathways included neutrophil extracellular trap formation, IL-17 signalling pathway, NOD-like receptor signalling pathway, NF-kappa β signalling pathway, B-cell receptor signalling pathway etc. (Fig. 5a). These pathways are closely related to the regulation of tumour immunity. Given that KEGG enrichment analysis tends to focus on genes with notable differences while ignoring some genes that are not greatly differentially expressed but have important biological functions, we adopted Gene Set Enrichment Analysis (GSEA) to screen important signalling pathways, which showed that Wnt/β-catenine was remarkably enriched in the NE group (Fig. 5b). Moreover, we performed differential analysis on the RNA_seq data of the two groups and found that the expression levels of WNT7A were evidently different (Fig. 5c). In order to further prove that NE can regulate the expression level of WNT7A, we detected the expression levels of Wnt1, Wnt2, Wnt3a, Wnt4, Wnt5a, Wnt6, Wnt7a and Wnt10a in tumour tissues of anti-PD-1 mAb + Vehicle and NE + anti-PD-1 mAb + Vehicle groups. The results showed that there were great differences in Wnt7a expression levels (Fig. 5d). Therefore, we speculated that NE regulates the secretion of CXCL9 and ADO in tumour cells through the WNT7A/β-catenin signalling pathway. To demonstrate the role of WNT7A in the regulation of CXCL9 and ADO secretion by NE in tumour cells, we transfected tumour cells with WNT7A-shRNA lentivirus or scramble-shRNA lentivirus (NC). Western blotting confirmed the efficiency of gene silencing (Fig. 5e). In A549^shNC^/H3122^shNC^ cells, NE significantly up-regulated the expression of WNT7A, β-catenin, CD39 and CD73, and downregulated the expression of CXCL9; however, in A549^shWNT7A^/H3122^shWNT7A^ cells, NE did not make corresponding regulation of these proteins (Fig. 5f). The results indicated that NE exerted its effects by regulating the expression of WNT7A. β-Catenin is a key downstream effector molecule in the Wnt signal transduction pathway and a hub protein that activates the downstream pathway [29]. In order to investigate the role of β-catenin in the regulation of CXCL9 and ADO secretion by NE, we transfected tumour cells with β-catenin-shRNA lentivirus or scramble-shRNA lentivirus (NC). Western blotting was used to confirm the efficiency of gene silencing. In A549^shNC^/H3122^shNC^ cells, NE/WNT7A significantly up-regulated the expression of WNT7A, β-catenin, CD39 and CD73, and downregulated the expression of CXCL9; however, in A549^shβ-catenin^/H3122^shβ-catenin^ cells, NE and WNT7A did not regulate β-catenin, CD39, CD73 or CXCL9 accordingly (Fig. 5g). These results indicate that β-catenin plays a crucial role in the regulation of NE. In addition, the results of the immunohistochemical analysis showed that compared with the anti-PD-1 mAb + Vehicle group, the expression of β-catenin in the anti-PD-1 mAb + NE group was significantly increased (Fig. 5h). These data suggest that NE regulates CXCL9 and ADO secretion in tumour cells through the WNT7A/β-catenin signalling pathway.

### Propranolol (Prop), a β2-receptor antagonist, reduces NE-induced anti-PD-1 mAb resistance

**Fig. 6:**
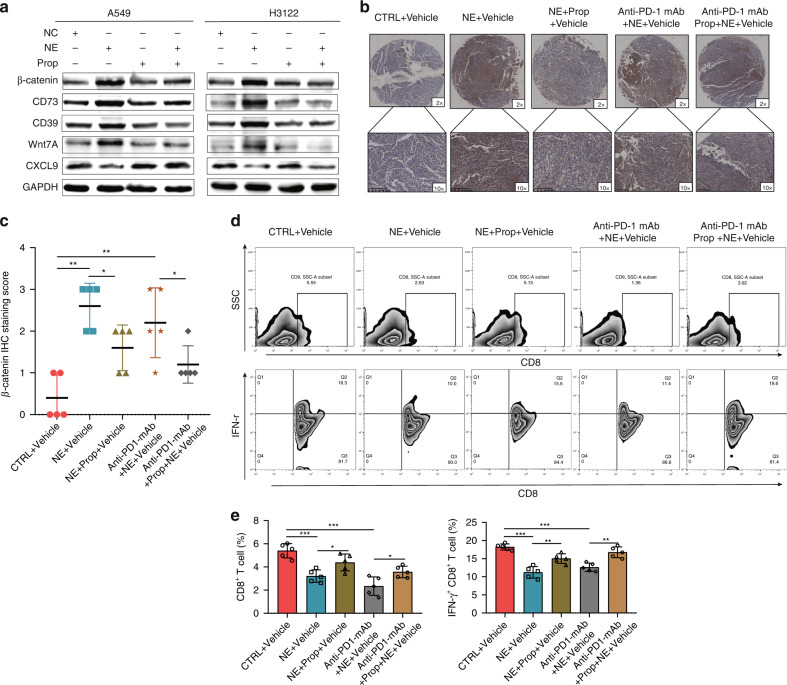
Propranolol (Prop), a β2-receptor antagonist, reduces norepinephrine (NE)-induced anti-PD-1 mAb resistance. **a** Prop can antagonise NE-induced β-catenin, CD39 and CD73 expression, as well as NE-inhibited CXCL9 expression in lung adenocarcinoma (LUAD) cells; the expression levels of β-catenin (**b**, **c**), the proportion of CD8^+^ T cells and the IFN-γ^+^ CD8^+^ T-cell rate (**d**, **e**) in the tumours of CTRL + Vehicle, NE + Vehicle, NE + Prop + Vehicle, anti-PD-1 mAb + NE + Vehicle and anti-PD-1 mAb + NE + Prop + Vehicle groups. *P < 0.05, **P < 0.01.

Previous studies have shown that NE is mainly involved in the regulation of the tumour immune microenvironment through β2-adrenoceptors (β2-AR) [30–34]. In addition, Prop, one of the most common β2-receptor antagonists, has been used in several studies to block β-adrenergic signalling [31, 35, 36]. Therefore, we speculated that Prop can antagonise the regulation of CXCL9 and ADO secretion by NE, thereby improving NE-induced anti-PD-1 mAb resistance. Firstly, cell experiments were performed to verify that NE could significantly up-regulate the expression of WNT7A, β-catenin, CD39 and CD73, and downregulate the expression of CXCL9; however, after adding Prop, NE could not regulate these proteins accordingly (Fig. 6a). We further compared the tumour size of CTRL + Vehicle, NE + Vehicle and NE + Prop + Vehicle groups, which showed that Prop could antagonise the effect of NE on tumour progression (Fig. 1d, e). Moreover, by comparing the tumour size of anti-PD-1 mAb + Prop + Vehicle and anti-PD-1 mAb + Vehicle groups, anti-PD-1 mAb and Prop combined therapy showed better therapeutic effects than anti-PD-1 mAb alone (Fig. 1d, e). By comparing the expression levels of β-catenin (Fig. 6b, c), the proportion of CD8^+^ T cells and the rate of IFN-γ^+^ CD8^+^ T cells (Fig. 6d, e) in CTRL + Vehicle, NE + Vehicle, NE + Prop + Vehicle, anti-PD-1 mAb + NE + Vehicle and anti-PD-1 mAb + NE + Prop + Vehicle tumours, we found that Prop antagonised NE to up-regulate β-catenin expression and inhibit CD8^+^ T-cell infiltration and function. These results all suggested that Prop reduced NE-induced anti-PD-1 mAb resistance.

## Discussion

Mental stress is associated with the immunosuppressed state of the body, which increases patients’ susceptibility to malignancy and infection, and can also result in poor response to treatment [37, 38]. Previous studies have confirmed a possible mechanism whereby negative psychological emotions reduce immune function in tumour patients; psychological events stimulate a series of non-specific reactions, leading to neurotransmitter secretion disorders, and ultimately reducing the body’s immune function [39, 40]. The above studies all show that mental stress is not only closely related to the immune disorder but also can regulate the immune function by affecting the secretion of neurotransmitters. Therefore, a better understanding of the potential functions of neurotransmitters in tumorigenesis, immunity and inflammation is expected to develop novel anti-tumour therapies, while facilitating synergistic immunotherapy. In this finding, we explain the relationship between CNS disorders and anti-PD-1 mAb in LUAD patients, which further emphasises the influence of stress, depression and other mental factors on tumour patients. In addition, the study confirms the broad prospect of CNS drug-combined immunotherapy for tumour treatment.

The application of anti-PD-1 mAb is a milestone in immunotherapy. Although anti-PD-1 mAbs have demonstrated impressive efficacy in the treatment of solid tumours, durable sensitivity occurs in only a small subset of patients. Therefore, it is of great significance to study the mechanism of anti-PD-1 mAb resistance to improve the clinical effect of immunotherapy and the life quality of patients with tumours. The causes of anti-PD-1 mAb resistance include insufficient tumour immunogenicity, irreversible T-cell depletion, immunosuppressive microenvironment, oncogene mutations etc [22]. It is worth noting that neurotransmitters can affect the immune system in multiple ways, leading to the immune escape of tumours [12]. An animal model study demonstrated that stress could elevate plasma corticosterone levels and up-regulate the expression of glucocorticoid-inducible factor Tsc22d3, which blocked type I IFN responses in dendritic cells and IFN-γ^+^ T-cell activation [41]. In addition, neurotransmitters such as cortisone, NE and epinephrine can inhibit the killing of tumour cells by monocytes [42]. Therefore, in this study, we explored the relationship between neurotransmitters and anti-PD-1 mAb resistance. We showed that increased levels of NE in LUAD patients could decrease the secretion of chemokine CXCL9 and disturb the immunosuppressive metabolite ADO in tumour cells, thereby reducing the proportion and function of CD8^+^ T cells in the tumour microenvironment, resulting in anti-PD-1 mAb resistance.

Interestingly, we found that the WNT7A/β-catenin signalling pathway plays a crucial role in NE’s aforementioned actions. Since the discovery of the first member of the Wnt family in 1982, research on Wnt signalling have steadily increased. The Wnt/β-catenin signalling pathway is required for embryonic development and adult tissue homoeostasis regeneration, whose abnormalities are closely related to the occurrence and progression of many diseases [43]. A large number of clinical and basic experiments have shown that the members of Wnt family, including WNT1, WNT2, WNT3A etc., are highly expressed in malignant tumour tissues, which can enhance tumour occurrence, growth and metastasis [44, 45]. A previous study also showed that NE-stimulated hepatic stellate cells secreted frizzled-related protein 1 to promote HCC progression following chronic stress via augmentation of a Wnt16B/β-catenin positive feedback loop [46]. In summary, the Wnt/β-catenin signalling pathway is dysregulated at nearly all stages of tumorigenesis, from malignant transformation to metastasis, spread and drug resistance. Especially, the Wnt/β-catenin signalling pathway can also disrupt tumour immune surveillance, promoting immune escape and resistance to immunotherapy [22, 47]. A series of studies have linked the abnormal activation of Wnt signalling with tumour development, drug resistance and immune escape. β-catenin promotes tumour development by regulating the expression of immune escape-related molecules (CCL4, CD47 and PD-L1) through transcription factors (MYC, TCF/LEF, NF-κβ, SNAI1, etc.) [48, 49]. Activation of the Wnt/β-catenin pathway in metastatic melanoma can ultimately lead to the reduction of T-cell infiltration. The main mechanism of this pathway is through the activation of recombinant activating transcription factor 3 (ATF3), which inhibits the expression of CCL4 and reduces the recruitment of CD103^+^ dendritic cells, ultimately affecting the activation of CD8^+^ T cells and limiting the efficacy of checkpoint inhibitors [50]. Through single-cell sequencing of clinical samples from more than 1000 colon cancer patients, one study found that 85% of MSI-H colon cancer patients did not benefit from immunotherapy because of the abnormal transduction of Wnt/β-catenin signalling. With the upregulation of WNT/β-catenin signalling, immune cell infiltration in colon cancer was reduced, suggesting that Wnt/β-catenin signalling is inversely correlated with T-cell infiltration [51]. These studies suggest that the Wnt/β-catenin signalling pathway takes part in the regulation of the tumour immune microenvironment. Based on these reports, this finding further confirms the great role of the WNT7A/β-catenin signalling pathway in the formation of immunosuppressant microenvironments.

**Fig. 7:**
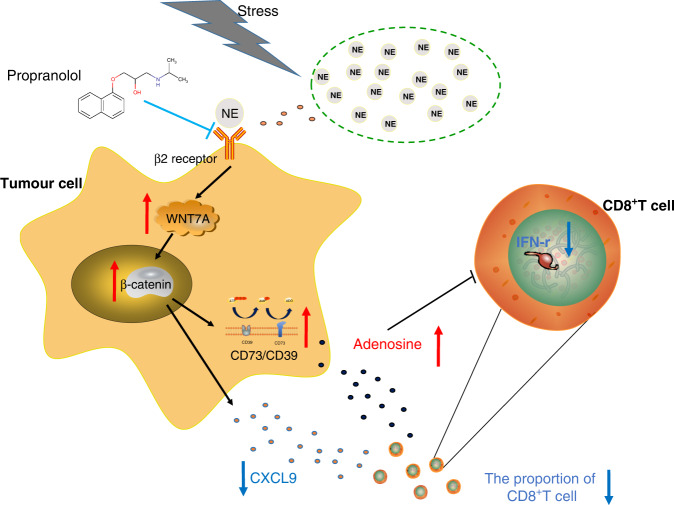
A general overview of the mechanism. The schematic diagram of the proposed mechanism by which NE induces anti-PD-1 mAb resistance.

In this study, we analysed the levels of neurotransmitters in the plasma of anti-PD-1 mAb-resistance patients and sensitive patients, which was further verified in animal models; as a result, we found that NE can cause anti-PD-1 mAb resistance. Importantly, this study shows that NE can affect the secretion of the chemokine CXCL9 and the immunosuppressive metabolite ADO in tumour cells by regulating the WNT7A/β-catenin signalling pathway, thereby reducing the chemotaxis and function of CD8^+^ T cells, resulting in anti-PD-1 mAb resistance. More importantly, propranolol could improve the NE-induced anti-PD-1 mAb resistance (Fig. 7). This finding sheds new light on the causes of anti-PD-1 mAb resistance in lung adenocarcinoma patients, which provides a new strategy for tumour treatment. The combination of CNS drugs and immunosuppressants has a broad application prospect in improving the life quality and survival rate of tumour patients.

## Supplementary information

Supplementary material legends

Supplementary figure 1

Supplementary figure 2

Supplementary figure 3

Supplementary Table 1

Supplementary Table 2

aj-checklist

## Data Availability

All data generated or analysed during this study are included in this published article (and its supplementary information files). Raw data are available from the corresponding author upon request.
