# Supplementary material for: Norepinephrine inhibits CD8+ T-cell infiltration and function, inducing anti-PD-1 mAb resistance in lung adenocarcinoma
Source: Br J Cancer. 2023 Jan 16;128(7):1223–35. doi: 10.1038/s41416-022-02132-7 (PMC10050078; doi:10.1038/s41416-022-02132-7)
Supplement: Supplementary file 1 — Supplementary material legends [file 41416_2022_2132_MOESM1_ESM.docx]

**Supplementary figure 1** The quantification of CD8^+^ T cell migration activity was measured and compared. (A) CD8^+^ T cells treated with medium containing the same amount of NE; (B) Western blotting confirmed the efficiency of gene silencing; (C) CD8^+^ T cells treated with the supernatants from NC/NE-treated A549^shCXCL9/shNC^/H3122^shCXCL9/shNC^.

**Supplementary figure 2** The PCA plot and the OPLS-DA model of the anti-PD-1 mAb group vs. the anti-PD-1 mAb + NE group. (A) The PCA plot of anti-PD-1 mAb and anti-PD-1 mAb + NE groups in positive ion mode; (B) the PCA plot of different groups in negative ion mode; The OPLS-DA plot of the two groups in positive ion mode(C) and negative ion mode (F); the permutations test of the two groups in positive ion mode (D) and negative ion mode (G); the VIP plot of two groups in positive ion mode (E) and negative ion mode (H).

**Supplementary figure 3** The IFN-γ function of CD8^+^ T cells was measured and compared in PBMCs. (A) PBMCs treated with medium containing the same amount of NE; (B) PBMCs treated with the supernatants from NC/NE-treated A549^shNC^/H3122^shNC^.

**Supplementary Table 1** The expression levels of common chemokines and receptors in anti-PD-1 mAb + Vehicle and NE + anti-PD-1 mAb + Vehicle, which were detected by PCR array.

**Supplementary Table 2** The differential metabolites were obtained by differential analysis, whose VIP value is more than 1.2.
