# Supplementary figures and images for: Norepinephrine inhibits CD8+ T-cell infiltration and function, inducing anti-PD-1 mAb resistance in lung adenocarcinoma
Source: Br J Cancer. 2023 Jan 16;128(7):1223–35. doi: 10.1038/s41416-022-02132-7 (PMC10050078; doi:10.1038/s41416-022-02132-7)

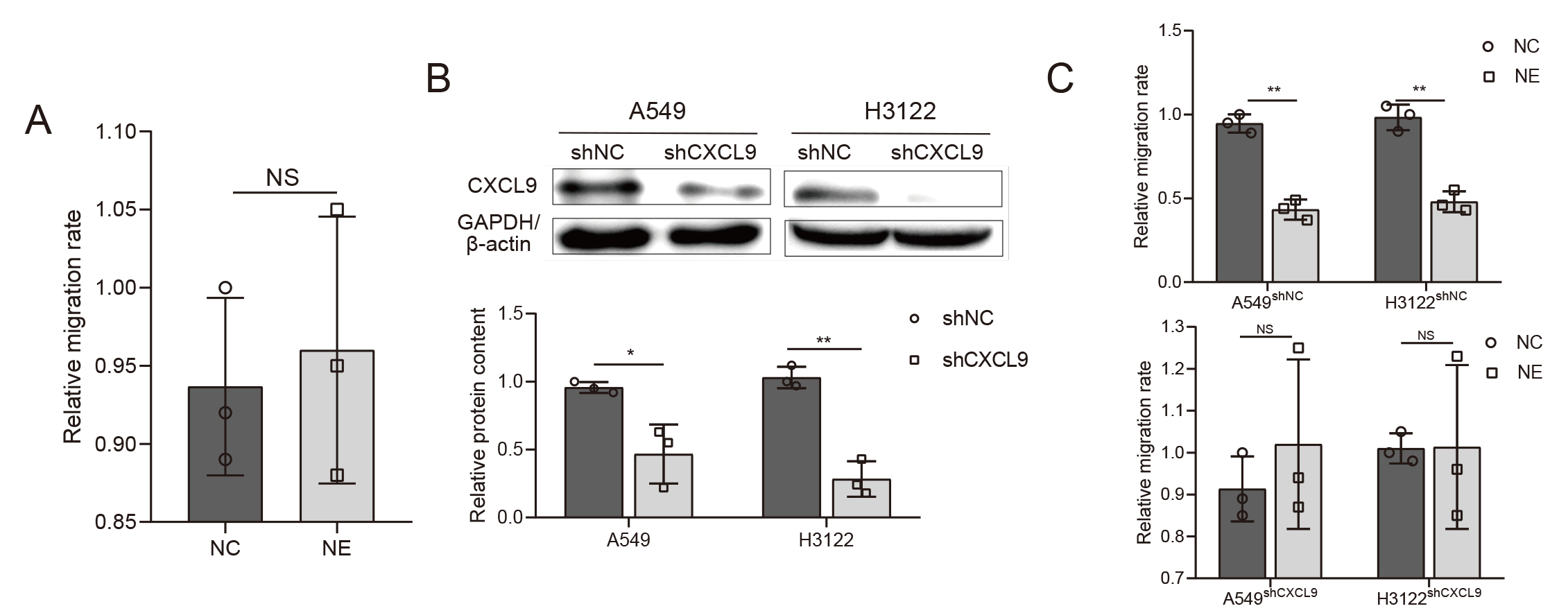

Supplement: Supplementary file 2 — Supplementary figure 1 [file 41416_2022_2132_MOESM2_ESM.tif]

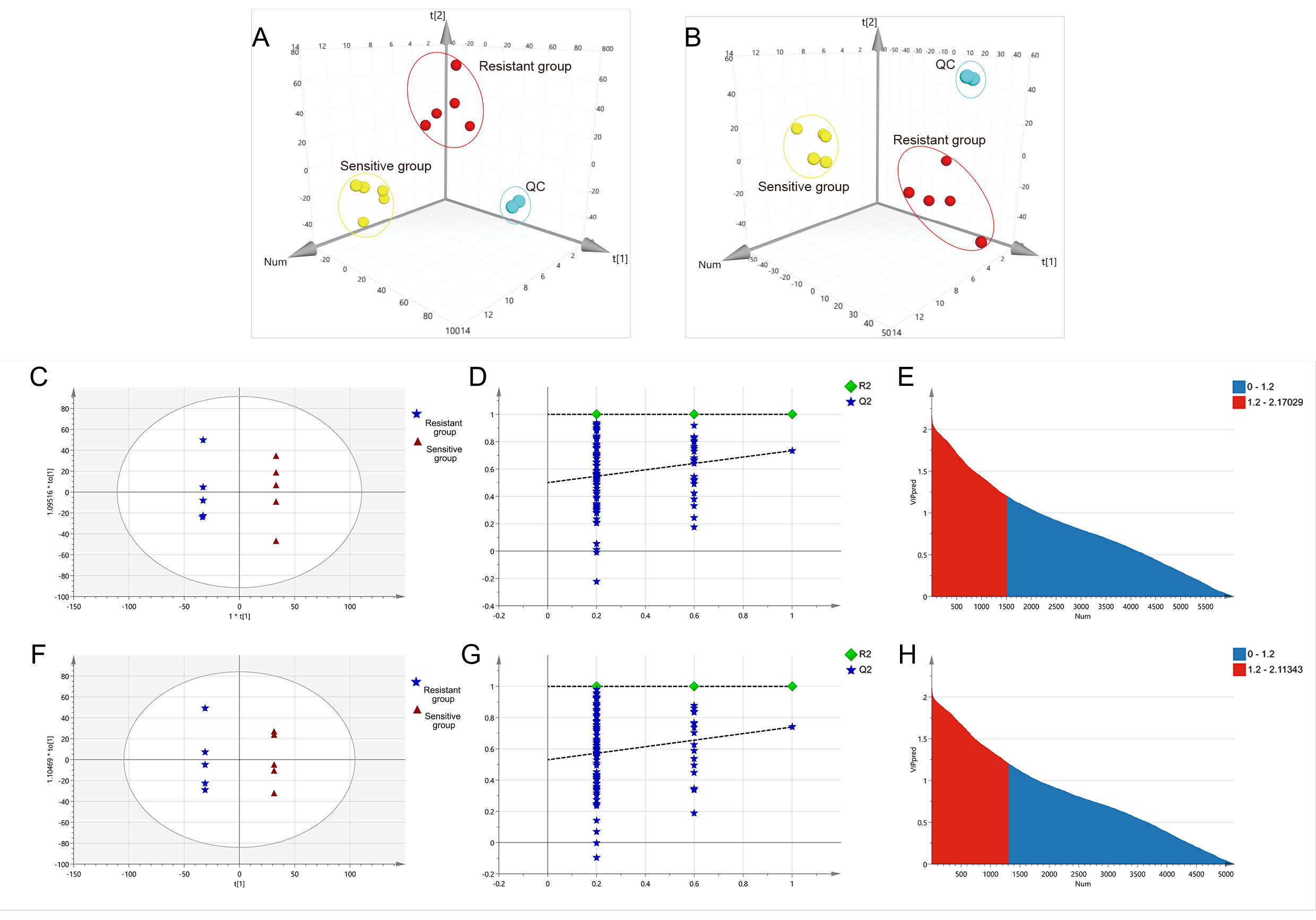

Supplement: Supplementary file 3 — Supplementary figure 2 [file 41416_2022_2132_MOESM3_ESM.tif]

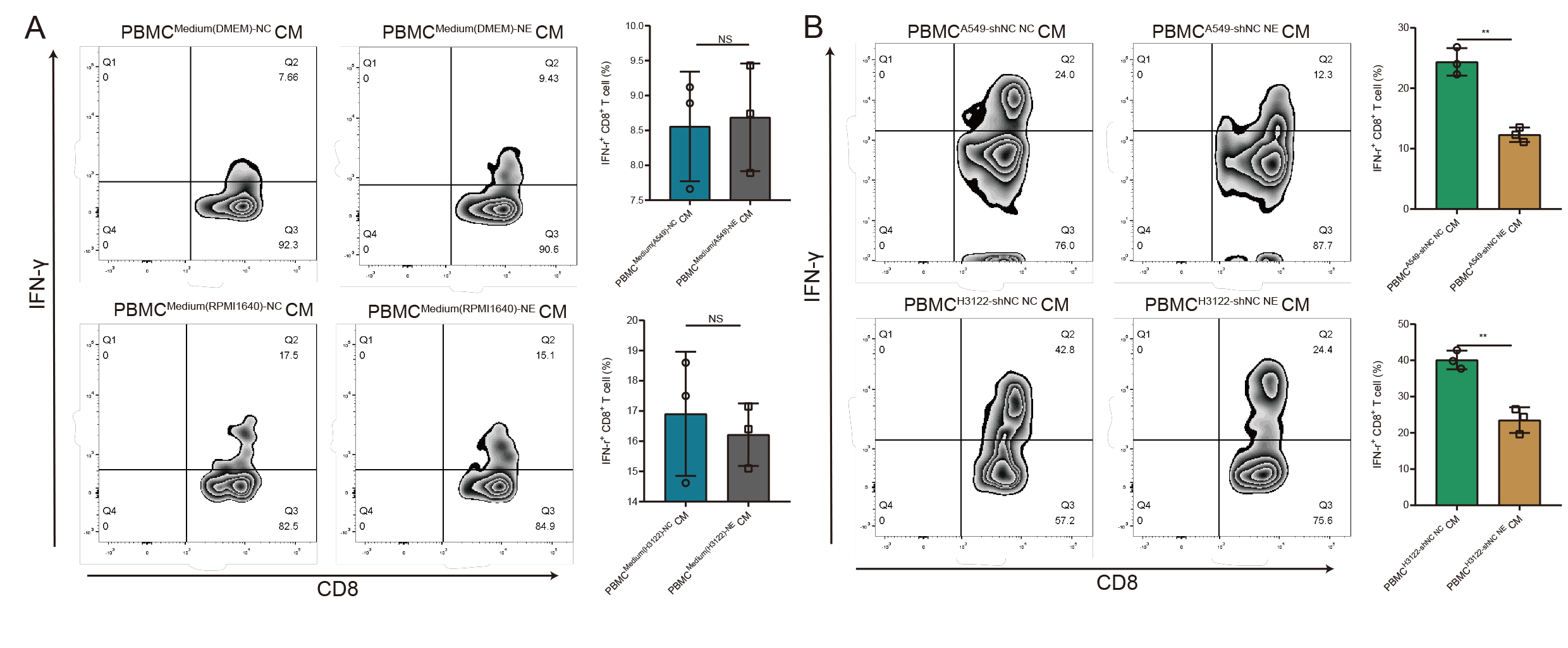

Supplement: Supplementary file 4 — Supplementary figure 3 [file 41416_2022_2132_MOESM4_ESM.tif]
